# Developing 3D Organoid Raft Cultures from Patient-Derived Xenografts as Rapid Models to Screen Efficacy of Experimental Therapeutics

**DOI:** 10.3390/ijms232214392

**Published:** 2022-11-19

**Authors:** Prachi Bajpai, Nilam Sanjib Banerjee, Dianne W. Moore, Hyung-Gyoon Kim, Farrukh Afaq, Carlo M. Contreras, Martin J. Heslin, Vishnu B. Reddy, Shajan Peter, Sooryanarayana Varambally, Sameer Al Diffalha, Upender Manne

**Affiliations:** 1 Department of Pathology, University of Alabama at Birmingham, Birmingham, AL 35233, USA; 2 Department of Biochemistry and Molecular Genetics, University of Alabama at Birmingham, Birmingham, AL 35233, USA; 3 Department of Surgery, Ohio State University, Columbus, OH 43210, USA; 4 Mitchell Cancer Institute, University of South Alabama, Mobile, AL 36688, USA; 5 O’Neal Comprehensive Cancer Center, University of Alabama at Birmingham, Birmingham, AL 35233, USA; 6 Division of Gastroenterology, Department of Medicine, University of Alabama at Birmingham, Birmingham, AL 35233, USA

**Keywords:** organoid raft cultures (ORCs), patient-derived xenografts (PDX), colon cancer, navitoclax (ABT-263), 3-dimensional (3D) culture model

## Abstract

Reliable preclinical models are needed for screening new cancer drugs. Thus, we developed an improved 3D tumor organoid model termed “organoid raft cultures” (ORCs). Development of ORCs involved culturing tumors ex vivo on collagen beds (boats) with grid supports to maintain their morphological structure. The ORCs were developed from patient-derived xenografts (PDXs) of colon cancers excised from immune-deficient mice (NOD/SCID/IL2Rgamma^null^). We utilized these new models to evaluate the efficacy of an investigational drug, Navitoclax (ABT-263). We tested the efficacy of ABT-263, an inhibitor of BCL-2 family proteins, in these ORCs derived from a PDX that showed high expression of antiapoptotic BCL2 family proteins (BCL-2, BCL-X_L_, and BCL-W). Hematoxylin and eosin staining evaluation of PDXs and corresponding ORCs indicated the retention of morphological and other histological integrity of ORCs. ORCs treated with ABT-263 showed decreased expression of antiapoptotic proteins (BCL2, BCL-X_L_ and BCL-W) and increased proapoptotic proteins (BAX and PUMA), with concomitant activation of caspase 3. These studies support the usefulness of the ORCs, developed from PDXs, as an alternative to PDXs and as faster screening models.

## 1. Introduction

In the process of drug development, cell-based preclinical models are useful in early phases of drug testing. Currently, 3-dimensional (3D) organoids are routinely used in early phases of drug discovery and validation studies. Recent studies have also developed patient-derived tumor organoids (tumor cells derived from individual patients) to assess therapy responses to personalize cancer treatments [1,2,3,4]. However, 3D organoid models that recapitulate human tumor morphology, genetic backgrounds, and stroma (both human and experimental host), which are represented in PDXs, and that can be used to screen efficacy of experimental therapeutic agents in preclinical setting are lacking. Thus, we developed 3D organoid raft culture (ORC) models from colon cancer patient-derived xenograft (PDX) tissues to determine the feasibility and value of their utilization in testing the efficacy of an investigational new drug (IND), Navitoclax (ABT-263), an inhibitor of Bcl-2 family proteins.

Current treatment guidelines for CRC include use of 5-fluorouracil (5FU)/leucovorin (LV)/oxaliplatin/irinotecan for advanced stage disease [5,6], and they do not allow for individualized therapy. Although, for metastatic CRCs (mCRCs), some targeted biologic therapies are available (e.g., cetuximab therapy for mCRCs if they express EGFR and exhibit wild-type *K-RAS*, *N-RAS*, and *B-RAF*), overall survival rates remain poor (<15%) [7,8]. Thus, there is a need to develop biomarker-based therapies and precision targeting for CRC. Like in other cancers, in CRC, the Bcl-2 proteins, which facilitate apoptosis, are dysregulated and contribute to tumor growth, progression, and therapy resistance [9]. For cellular homeostasis, cells go through a form of programmed cell death termed “apoptosis”. Tumors are initiated when this equilibrium between proliferation and apoptosis is perturbed. In this context, several molecular abnormalities are being studied; one such event is high expression of the antiapoptotic protein, BCL-2. This oncoprotein leads to tumor progression, [10,11], particularly in reference to CRC [12], and promotes resistance to targeted therapies (against the RAS–RAF–MAPK pathway) [13,14]. It is thus plausible to speculate that interfering with antiapoptotic machinery would improve the efficacy of other therapeutic agents. Navitoclax, a synthetic small molecule, is orally bioavailable, has antineoplastic activity, and functions as an antagonist of antiapoptosis proteins [15], which include the BCL-2 family members, BCL-X_L_, BCL-2, and BCL-W [16].

The current study focused on developing 3D ORC models for CRC by utilizing PDXs that exhibited high expression of BCL-2 family proteins (ORC-82) to evaluate the usefulness of these models in assessing the efficacy of ABT-263. The ORC models will aid in culturing human tumors ex vivo on collagen beds/boats with grid supports to maintain their tumor heterogeneity, morphology, and molecular makeup.

## 2. Results

### 2.1. Organoid Raft Cultures Retain the Morphological Features of Colorectal Cancer

**Figure 1:**
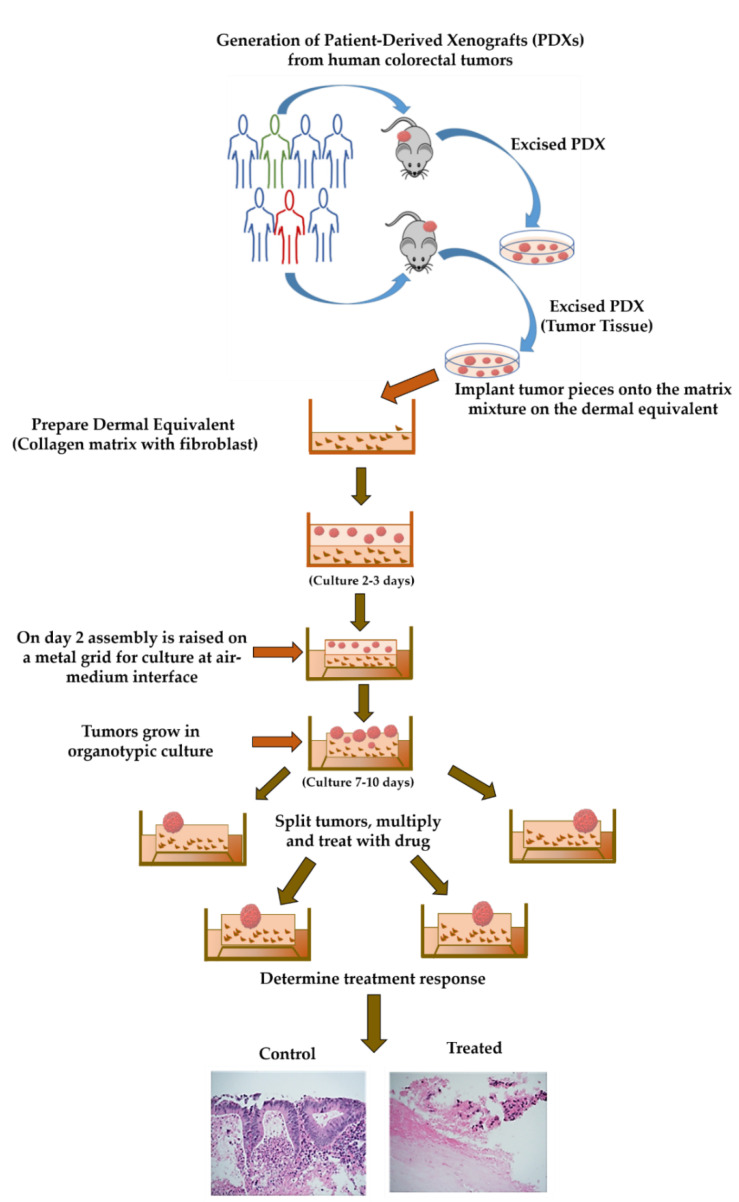
Schematic representation to generate organoid raft cultures from primary CRCs.

**Figure 2:**
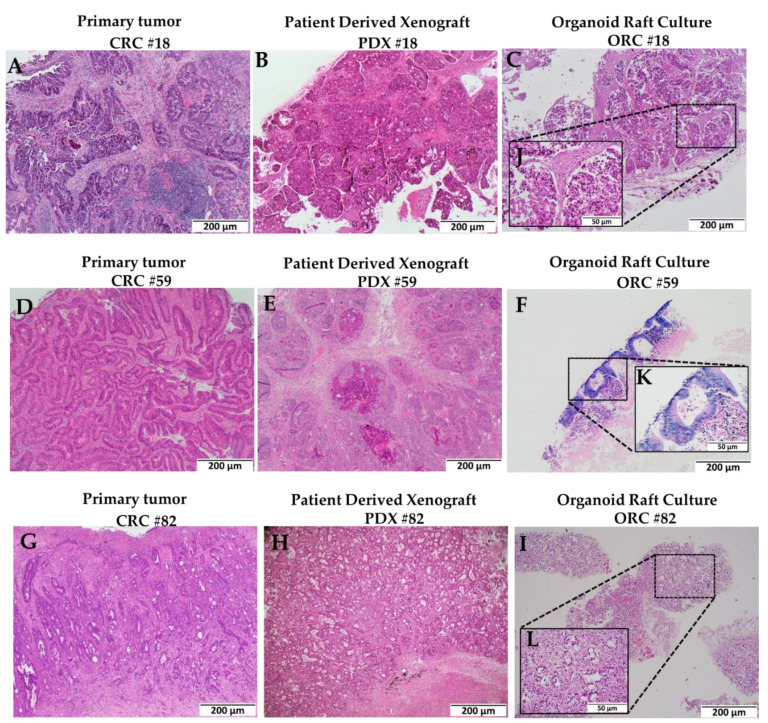
Morphological features were well retained from primary tumor to patient-derived xenografts (PDXs) to organoid raft cultures (ORCs). Hematoxylin and eosin (H&E) images at 4× magnification (scale bar: 200 µm) in (**A**–**I**) and panels (**J**–**L**), which are shown in the insets with dotted lines and captured at 20× magnification to showcase glandular morphology of colorectal cancers (CRCs) (scale bar: 50 µm). Panels (**A**,**D**,**G**) are primary tumors (CRCs); panels (**B**,**E**,**H**) are PDXs; and panels (**C**,**F**,**I**) are ORCs derived from three different CRC patients.

To illustrate the usefulness of the methodology, described in Figure 1, the organoids generated from PDXs retained their cell morphology. To demonstrate retention of the original morphological features, we stained formalin-fixed, paraffin-embedded (FFPE) sections from primary CRC tissues collected from patients in a PDX model that was used to generate ORCs and from the established ORCs with hematoxylin and eosin (H&E). The structural morphology of these primary CRC tissues collected from three patients (CRC #18, CRC #59, and CRC #82) were well persevered when the tissue was used to generate PDXs (PDX #18, PDX #59, and PDX #82) and subsequently organoid raft cultures ORCs (ORC #18, ORC #59, and ORC #82) (Figure 2A–I, 4× magnification). The higher magnification images of ORCs demonstrating the glandular morphology of colon adenocarcinoma were retained from its original primary tumor (inset, Figure 2J–L). Cellular morphology was distinct and well conserved as we propagated the primary tumor tissues to PDXs and then to ORCs (Figure 2). The glandular structures, arranged in their characteristic parallel tube-like pattern, were supported by loose connective tissue (Figure 2). This conventional characteristic of adenocarcinomas was retained along with other morphologic features, indicating the usefulness of ORCs.

### 2.2. Colorectal Primary Tissues Express Varying Levels of BCL-2 Family Proteins

**Figure 3:**
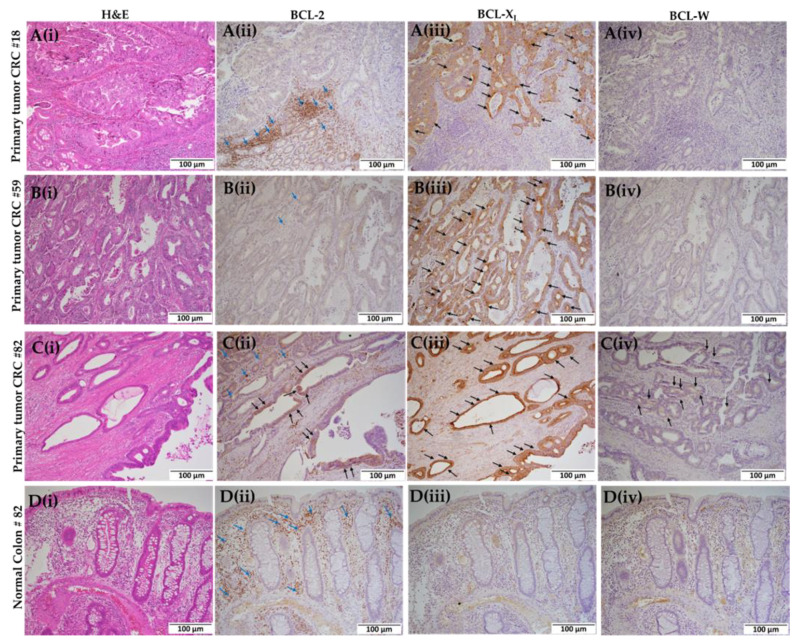
Immunohistochemical detection of BCL-2 family proteins in primary tumors of colorectal cancers (CRC) are shown in panels (**A**–**C**) and in the matching normal colonic epithelium of case #82 as illustrated in panel (**D**). Panel (**A**) illustrates the staining in tumor tissue of CRC#18. (**A**(**i**)) Hematoxylin and eosin (H&E) staining, (**A**(**ii**)) BCL-2, (**A**(**iii**)) BCL-XL, and (**A**(**iv**)) BCL-W. Panel (**B**) illustrates the staining in tumor tissue of CRC#59. (**B**(**i**)) H&E staining, (**B**(**ii**)) BCL-2, (**B**(**iii**)) BCL-XL, and (**B**(**iv**)) BCL-W. Panel (**C**) illustrates the staining in tumor tissue of CRC#82. (**C**(**i**)) H&E staining, (**C**(**ii**)) BCL-2, (**C**(**iii**)) BCL-XL, and (**C**(**iv**)) BCL-W (patchy weak staining). Panel (**D**) shows the staining of normal colonic epithelium from CRC#82 case. (**D**(**i**)) H&E staining, (**D**(**ii**)) BCL-2, (**D**(**iii**)) BCL-XL, and (**D**(**iv**)) BCL-W. No immunostaining is noted in normal colonic epithelial cells Panel (**D**(**ii**–**iv**)). All images are taken at 10× magnification (scale bar: 100 µm), and arrows (black) indicate positivity of the immunogenic signal in cancer cells. The immunogenic signal in lymphocytes serves as internal positive controls for Bcl-2 staining (blue arrows).

All three colorectal primary tissues (CRC#18, CRC#59, and CRC#82) were analyzed for BCL-2 family proteins by immunohistochemistry (Figure 3A–C). The tumor tissue in CRC#18 expressed a high amount of BCL-X_L_ (Figure 3A(iii)) but showed no expression of BCL-2 (Figure 3A(ii)) and BCL-W (Figure 3A(iv)). The tumor tissue of CRC#59 also expressed high BCL-X_L_ (Figure 3B(iii)) but showed low expression of BCL-2 (Figure 3B(ii)) and no expression BCL-W (Figure 3B(iv)). The tumor tissue of CRC-82 expressed high levels of BCL-2 (Figure 3C(ii)) and BCL-X_L_ (Figure 3C(iii)), but a modest patchy expression pattern was noted for BCL-W (Figure 3C(iv)). Since CRC-82 had relatively higher expression of BCl-2 and BCL-X_L_ with modest BCL-W expression, it is a good model to develop PDXs and to generate organoids (ORC-82) to evaluate the efficacy of ABT-263. None of the Bcl-2 family proteins were expressed in the matched normal tissues of CRC-82 (Figure 3D(ii–iv)). The clinicopathologic characteristics of all three CRCs used in the study are summarized in Table 1.

**Figure 4:**
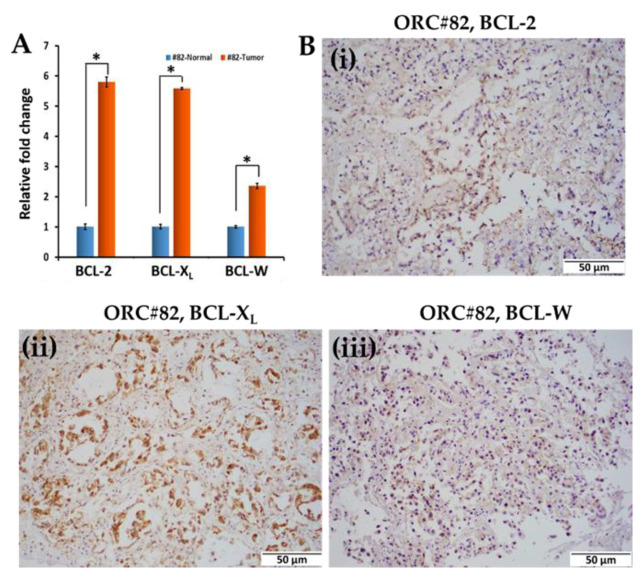
Gene expression in CRC-82 and immunophenotypic levels of antiapoptotic molecules in the organoids generated. (**A**) BCL-2, BCL-X_L_, and BCL-W in primary human CRCs (#82-Tumor) and its corresponding normal tissue (#82-Normal). (**B i**–**iii**) Immunohistochemical staining of BCL-2, BCL-X_L_, and BCL-W in organoids (ORC-82). All images are taken at 20× magnification (scale bar: 50 µm). The statistical significant values *p* ≤ 0.05 are denoted by asterisk *.

Further, tumor and normal tissues of CRC-82 were analyzed for RNA expression of *BCL2*, *BCL-X_L_*, and *BCL-W* by RT-PCR. CRC-82 exhibited 5.8-, 5.6-, and 2.4-fold RNA expression of these molecules, relative to its normal tissue control (Figure 4A). These results correlate well with the immunophenotypic expression of BCl-2 and BCL-X_L,_ and BCL-W as shown in Figure 3C. After the generation of ORC-82, expression of BCL-2 family proteins was confirmed by immunohistochemistry (Figure 4B i–iii).

### 2.3. Effect of ABT-263 Treatment on BCL-2 Family Proteins in Organoid Raft Cultures

**Figure 5:**
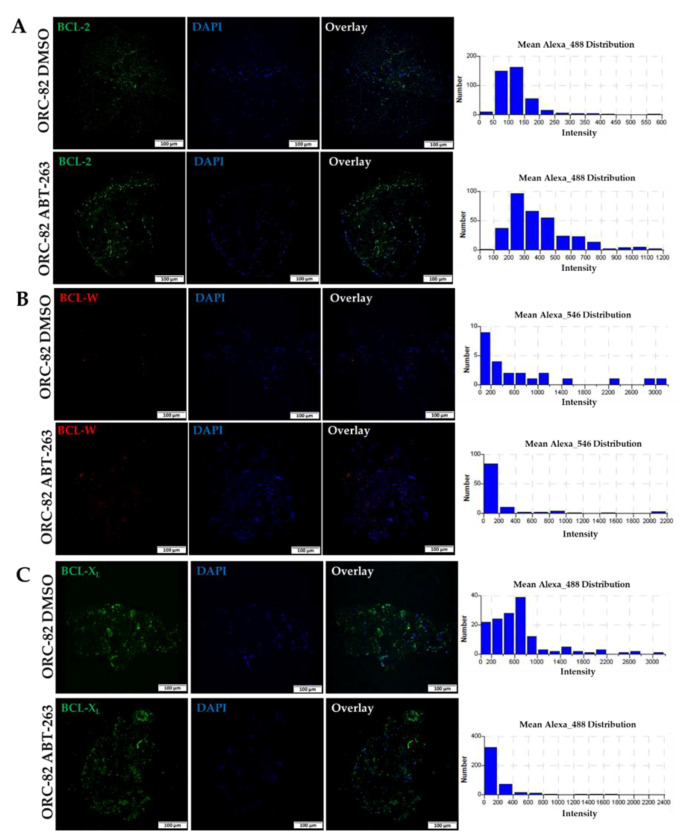
Immunofluorescence staining of antiapoptotic molecules in organoid OCR-82 after ABT-263 treatment. Panel (**A**) shows expression of BCL-2 (green); panel (**B**) shows expression of BCL-W (red); and panel (**C**) shows expression of BCL-X_L_ (green) in DMSO control treatment (ORC-82-DMSO) and after ABT-263 treatment (ORC-82-ABT-263). Nuclei (blue) were stained by DAPI; quantification of immunofluorescence signals are represented in bar diagrams; and all images are at 10× magnification (scale bar: 100 µm).

ABT-263 functions as an antagonist of antiapoptotic proteins and has high affinity for BCL-2 antiapoptotic proteins, including BCL-2, BCL-X_L_, and BCL-W [17]. There are no reports on the expression status of *BCL-2* family members (mRNA levels) after treatment of CRC cells/organoids/PDXs with ABT-263. Thus, we analyzed, by immunofluorescence staining, the effect of ABT-263 (2.5 µM, for 72 h) on BCL-2, BCL-X_L_, and BCL-W proteins in ORC-82 and found no change in BCL-W, a modest decrease in BCL-X_L_, and a marginal increase in BCL-2 as compared to their respective controls (Figure 5A–C). Our findings for BCL-X_L_ are similar to those reported for human telomerase-immortalized retinal pigment epithelial cells [18] that the levels of BCL-X_L_ protein decrease after ABT-263 treatment (Figure 5A–C).

### 2.4. ABT-263 Treatment of Organoid Raft Cultures Decreases Cell Proliferation and Increases Apoptosis

**Figure 6:**
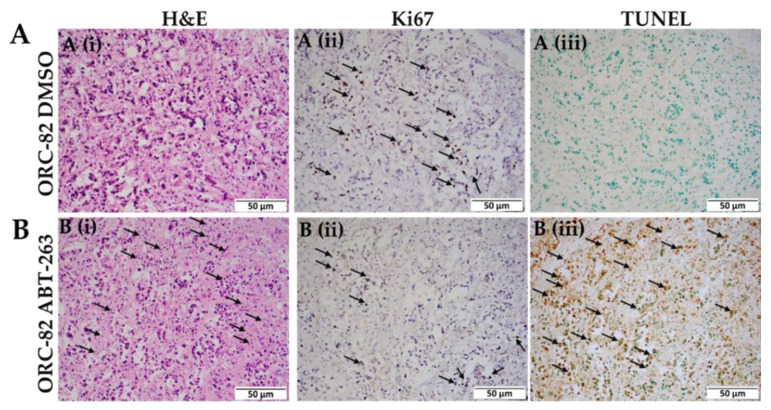
ABT-263 treatment induces apoptosis. Top panel (**A**) shows (**A**(**i**)) H&E, (**A**(**ii**)) Ki67 staining, and (**A**(**iii**)) TUNEL staining in ORC-82-DMSO, and bottom panel (**B**) shows (**B**(**i**)) H&E, (**B**(**ii**)) Ki67 staining, and (**B**(**iii**)) TUNEL staining after ABT-263 treatment (ORC-82-ABT-263). Arrows in (**B**(**i**–**iii**)) show high numbers of dying cells, and (**B**(**ii**)) shows reduced cell proliferation. All images at 20× magnification, (scale bar: 50 µm).

Since apoptosis is induced by BH3-only proteins either by antagonizing antiapoptotic proteins such as BCL-2 or by activating proapoptotic proteins [18,19], leading to activation of a caspase cascade [20,21], we thus first stained organoids (ORC-82) with H&E stain, for Ki67, and for TUNEL assays. The H&E staining showed high numbers of dying cells after ABT-263 treatment (Figure 6B(i)). Then we stained ABT-263-treated and DMSO-treated control ORC sections for Ki67 (marker for proliferation) and observed reduced Ki67 staining in ABT-263-treated ORCs (Figure 6B(ii)). Reduced numbers of Ki67-positive cells indicated that cell proliferation was reduced after ABT-263 treatment (Figure 6B(ii)) compared to DMSO control (Figure 6A(ii)). Further, the ABT-263 treatment induced apoptosis, as demonstrated by higher numbers of TUNEL-positive cells as compared to control (Figure 6B(iii)).

**Figure 7:**
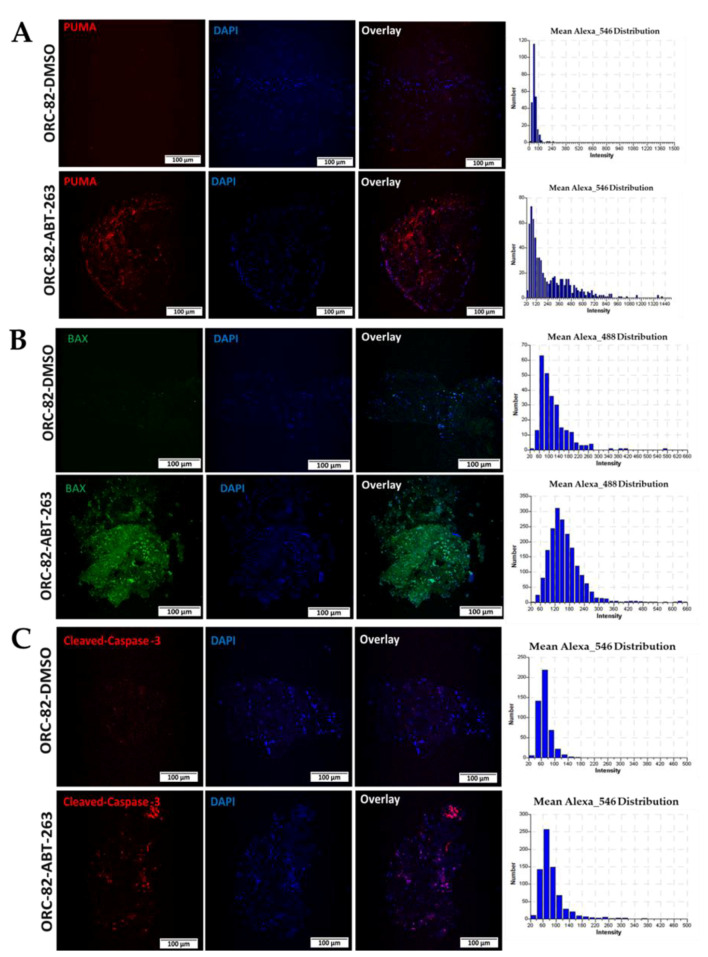
ABT-263 treatment in organoid raft cultures induces apoptosis by increasing PUMA and by activating BAX and cleaved-caspase 3. (**A**) Higher expression of PUMA (red staining), (**B**) BAX (green staining), and (**C**) cleaved-caspase 3 (red staining) were noted after ABT-263 treatment (ORC-82-ABT-263) as compared with DMSO control treatment (ORC-82-DMSO). The same sections that were presented in Figure 5 were costained for proapoptotic proteins, as described in methods Section 4.12. Nuclei (blue) were stained by DAPI; quantification of immunofluorescence signals are represented in bar diagrams; and all images are at 10× magnification (scale bar: 100 µm).

We also analyzed the effect of ABT-263 on proapoptotic BAX and on a BH3 domain-only protein, PUMA. After ABT-263 treatment, immunofluorescence images showed higher expression of PUMA (Figure 7A) and BAX (Figure 7B) along with activated caspase-3 (Figure 7C). This observation supports the conclusion that this strategy to propagate tumors in ex vivo conditions as 3D tumor models can be used to screen the efficacy of experimental drugs.

## 3. Discussion

Here, we describe a method for development of organoid raft cultures (ORCs) from patient-derived xenografts (PDXs) of colorectal adenocarcinomas (CRCs) and demonstrate its utility as a rapid drug-screening platform. Small pieces derived from PDXs were expanded rapidly (1 week–10 days) into ORCs. These ORCs maintained the tumor phenotype of the parent tumor and PDX models. This model retained the original histomorphology, and the method was highly reproducible. Similar findings are noted in our unpublished work on cervical cancer. The efficacy of an experimental drug, ABT-263, was tested within a week of ORC development. Formalin-fixed, paraffin-embedded sections of the ORCs were used to assess the effect of ABT-263 by evaluating the targeted molecules of this agent by qRT-PCR and proapoptotic proteins by immunofluorescence. This method is rapid, efficient, and economical compared to conventional in vivo xenograft models, yet it provides tissue/cellular- and molecular-level information compared to submerged cell culture methods.

PDXs are beneficial for experimental therapeutic studies because they are developed directly from human specimens and recapitulate the tumor microenvironment and molecular and cellular heterogeneity. However, utilizing PDXs for drug screening or to characterize the molecular targets is time-consuming and expensive. Therefore, the current study focused on developing rapid (one week to 10 days), cost-effective, and physiologically and molecularly relevant 3D-ORCs on collagen fiber boats to mimic the tumor human tissue-like environment.

The ORCs also have several advantages. One is that the physiologically relevant features of our ORCs, developed from PDX models, are retained because they are prepared on rafts that are composed of collagen and fibroblasts, which provide mechanical stability and support to growing cells by allowing their interactions with the matrix and allowing them to perform cellular functions in a tissue-like environment. An additional advantage is minimization of the effects of pericellular oxygen diffusion/concentration. In routine/ordinary cell culture systems, diffusion of molecular oxygen is a limiting factor (reviewed in [19]). For typical monolayer cultures, the distance of oxygen travel in the media by diffusion is longer than that for ORC models grown on collagen rafts. Indeed, in mammalian tissues, the oxygen diffusion distance is typically about 10–30 µm [20,21]. The distance traveled by diffused oxygen in culture media under routine cell culture conditions is presumably much lower at the bottom of the well/plate, although a precise measurement is not available [19]. However, the gradient oxygen concentration is about 100 fmol/h/cell at the bottom of the plate [22]. This is an important factor for overall functions of cells since the oxygen concentration is involved in gene expression, cell proliferation, and cell differentiation [23,24]. Furthermore, ORC models could be developed by modulating culturing/incubation conditions, for example, to study tumor-induced angiogenesis by co-culturing them with endothelial cells.

In summary, the ORCs are efficient models that maintain intratumoral heterogeneity and demonstrate their value for experimental therapeutic studies in conditions mimicking the physiological environment. Additionally, they provide an opportunity to develop ORCs from patients for screening therapeutic agents, particularly experimental drugs, before they are administered as part of clinical trials. This approach would help identify the best response among the wide array of drugs and save resources in terms of cost and time, without evaluating the efficacy of drugs in more expensive models or in patients. These models would also serve as potential tools to evaluate the concordance with the efficacy of drugs tested on organoids, PDXs, and the final clinical outcomes. It would be important to establish organoid biobanks, after annotating them for mutational and/or expression profiling for key molecular targets, and utilize them for evaluating the efficacy of drugs or therapeutic options available to treat cancers/diseases. Our proof-of-principle studies presented here provide details for the generation of ORCs and their utilization in drug testing. The ORCs would be a resource for faster screening of drugs with a path toward predicting the outcome of personalized therapy.

## 4. Materials and Methods

### 4.1. Materials Required for Organoid Raft Cultures (ORCs)

- Dulbecco’s modified Eagle’s medium (DMEM), supplemented with 10% fetal bovine serum and 1% Penicillin Streptomycin Antibiotic (100×, Corning, Cat # 30-002-CI, Fisher Scientific, Hampton, NH) was used for culturing feeder cells.
- We used Ham’s F12:F12 mix (1X) (1 L, package, GIBCO, Cat # 21700-0075, Thermo Fisher Scientific, Waltham, MA, USA). For reconstitution, directions on the package were followed, and 1.176 g of sodium bicarbonate was added. The pH was adjusted to 7.0 with 1 N HCl before filtration. F12, not more than a month old, should be used, as the pH fluctuates swiftly.
- Insulin stock (1000X) (Sigma–Aldrich, Cat # I-1882, Millipore Sigma, Burlington, MA, USA) was made by dissolving 100 mg in 20 mL sterile ultrapure water inside a cell culture hood. Aliquots were stored at −20 °C.
- Apo-transferrin stock (1000X) (Sigma–Aldrich, Cat # T1147, Millipore Sigma, Burlington, MA, USA) was made by dissolving 100 mg in 20 mL of sterile ultrapure water inside a cell culture hood. Aliquots were stored at −20 °C.
- Hydrocortisone-21-hemisuccinate stock (10,000X) (Sigma–Aldrich, Cat # H-488, Millipore Sigma, Burlington, MA, USA) was made by dissolving 100 mg in 25 mL of 100% ethanol inside a cell culture hood. Aliquots were stored at −20 °C.
- Human epidermal growth factor stock (10,000X) MilliporeSigma Calbiochem, Cat # 32-483-1200UG, Fisher Scientific, Hampton, NH, USA) was made by diluting 100 µL in 20 mL of sterile ultrapure water inside a cell culture hood. Aliquots were stored at −20 °C.
- Cholera toxin stock (10,000X, 1 mM) (Cayman Chemical, Cat # NC1425699, Fisher Scientific, Hampton, NH) was made by dissolving 1 mg in 2 mL of sterile ultrapure water inside a cell culture hood. Of this solution, 174 µL was added to 826 µL of sterile ultrapure water. Aliquots were stored at 4 °C. This solution should not be frozen.
- We used Type I Rat Tail Collagen (Corning, Cat # CB-40236, Fisher Scientific, Hampton, NH, USA).
- F12 stock (10X) (GIBCO, Cat # 21700-0075, Thermo Fisher Scientific, Waltham, MA, USA) was made by dissolving one 1-L package of Ham’s F12 medium in 100 mL of sterile ultrapure water. In this stock, pH was not adjusted, and sodium bicarbonate was not added. Filter-sterilized aliquots were then stored at −20 °C.
- We used phosphate-buffered saline (PBS) (10X) (Fisher BioReagents, Cat # BP39920, Fisher Scientific, Hampton, NH, USA).
- We used Antibiotic and Antimycotic mixture (Anti–Anti) (100X) (GIBCO, Cat # 15240062 Thermo Fisher Scientific, Waltham, MA, USA.
- We used 3T3-J2 mouse fibroblasts cells (Cat#EF303, Kerafast, Boston, MA, USA).
- Stainless steel grids (Edward J. Darby Inc, Philadelphia, PA, USA) measuring 1”× 1” (L × B) consisted of squares of wire mesh with square pores (size, 0.25 mm). The corners of the wired mesh were bent to make rafts/boats (approximate height 2 mm).

### 4.2. Generation of Patient-Derived Xenografts (PDXs)

After approval of the Institutional Review Board (IRB) of the University of Alabama at Birmingham (Animal Project Number (APN): IACUC-20207), colon cancer tissue for generation of patient-derived xenografts (PDXs) was collected. Histologically confirmed primary human tissue was obtained from the Department of Surgery at UAB and stored in DMEM with 10% fetal bovine serum. Tumors were cut into small pieces (0.5 × 0.5 × 0.5 cm), coated with Matrigel (BD 354234), and implanted subcutaneously into 8–10-week-old immune-deficient mice (NOD/SCID/IL2Rgamma^null^) within 1 to 4 h of resection of tumors. Once the tumor volume reached to 1500 mm^3^, mice were sacrificed, and tumors were harvested, cut into three pieces, and used as follows: (1) for generation of ORCs, (2) stored in freezing mix (mixture of 95% fetal bovine serum (FBS) and 5% DMSO) at −80 °C for future generation of PDXs, and (3) for FFPE block preparation.

### 4.3. Collection of the Patient-Derived Xenograft Tissue

After harvesting, PDX tissues were transferred to DMEM with 10% FBS and antibiotics, stored at 4 °C, and used within 4 h. Just before generation of ORCs, tissues were minced in aseptic conditions, suspended in 500 µL of FBS, and kept on ice until use.

### 4.4. Preparing Collagen Dermal Equivalent

J2 fibroblasts cells were harvested, and 5 million cells/mL were placed in FBS. In another tube, the following components were added and mixed by inverting:

- 400 µL of 10× Reconstitution Buffer (RB Buffer: 2.2 gm sodium bicarbonate, 0.2 gm sodium hydroxide (Sigma, Cat # 1310-73-2, Millipore Sigma, Burlington, MA, USA), and 4.76 gm HEPES (free acid; Sigma, Cat# H 3375) per 100 mL. Aliquots of 10 mL were stored at −20 °C);
- 400 µL 10× F12;
- 4 mL Type-1 Rat tail collagen.

To this mixture, 200 µL of J2 fibroblasts cells was added. This composition should be adjusted and multiplied to the number of wells one needs to make the collagen bed. Approximately, 750–800 µL per well was added in each 24-well plate. The plates were incubated for 15 min in a 37 °C CO_2_ incubator, so that the collagen along with other components set well.

### 4.5. Seeding on Collagen Bed/Boat

For the tumor pieces suspended in FBS (from Section 4.2), 50 µL was added to the collagen beds/boats in each well. The 24-well plate was incubated for 30 min, and then 1 mL of Raft Culture media with 2× Antibiotic Antimycotic (Sigma) was added.

### 4.6. Generation of Organoid Raft Cultures (ORCs)

After two to three days, the collagen beds were detached. Stainless steel grids (60 mm) were placed in 6-well plates, and detached collagen beds with tumor tissue were transferred onto them. Culture media (4 mL) was added, the culture was maintained for 10–20 days, and media was changed every alternate day (Figure 1).

### 4.7. Treatment with Navitoclax (ABT-263)

We tested Navitoclax (ABT-263) obtained from MedChem Express (Monmouth Junction, NJ, USA). ABT-263 inhibits BCL-2 family members BCL-X_L_, BCL-2, and BCL-W. Several PDXs in our repository were profiled for RNA and protein expression, and we selected a PDX model (PDX-82) that exhibited high levels of BCL2 family members. It was used to develop ORCs to test the effect of ABT-263. These ORCs were divided into groups treated with 2.5 µM of ABT-263 for 72 h or a DMSO-treated control.

### 4.8. Harvesting the Organoid Raft Cultures (ORCs)

To harvest the ORCs, media was aspirated, and cultures were rinsed with PBS. After detaching the steel grid, the entire assembly was submerged in 10% buffered formalin overnight. For better preservation of DNA, RNA, and proteins, use of buffered formalin is recommended. The next day, the assembled cassettes were transferred to 70% ethanol in specimen cups and kept at 4°C before embedding and sectioning. Superfrost/plus microscopic glass slides were used for better tissue adherence.

### 4.9. Immunohistochemical (IHC) Analysis

To evaluate expression of BCL-2, BCL- X_L_, and BCL-W, immunohistochemical (IHC) analysis was performed on ORC tissue sections as described earlier [12]. In brief, 5 µM thick FFPE sections were used. The sections were deparaffinized, rehydrated, and antigen-retrieved before incubating with BLOXALL (Cat. # SP-6000, Vector Laboratories, Burlingame, CA, USA) for 15 min to remove endogenous peroxidase activity. Tissue sections were then blocked with Normal Horse Serum Blocking Solution (Cat. # S-2012, Vector laboratories, Burlingame, CA, USA) for 1 h at room temperature and probed with specific antibodies for BCL-2 (Cat # M0887, Agilent Technologies, Santa Clara, CA, USA), BCL- X_L_ (Cat #2764, Cell Signaling Technology, Danvers, MA, USA), BCL-W (Cat #2724, Cell Signaling Technology, Danvers, MA, USA) and Ki67 (Cat # 790-4286, Roche, Indianapolis, IN, USA) protein for 1 h at room temperature. After three washes with 0.5% Tween in Tris-buffered saline (TBST), tissue sections were incubated for 1 h with ImmPRESS HRP Antimouse/Rabbit IgG (Peroxidase) (Cat. # MP-7402/MP-7401, Vector Laboratories, Burlingame, CA, USA) as a secondary antibody. After incubation, sections were subjected to washing in 0.5% TBST. DAB substrate (Cat. # SK-4105, Vector Laboratories, Burlingame, CA, USA) was used for color development. Slides were counterstained with hematoxylin solution (Cat. # H-3404, Vector laboratories, Burlingame, CA, USA), dehydrated, and mounted with VectaMount permanent mounting medium (Cat. # H-5000, Vector Laboratories, Burlingame, CA, USA).

### 4.10. RNA Extraction and Quantitative-PCR

The primary CRC tissues were divided into three equal fractions. One was frozen at −80 °C for protein assays; another was placed in 500 µL of Trizol and stored at −80 °C for RNA extraction; and the third was fixed overnight in 10% formalin, which was replaced with 70% ethanol and stored at 4 °C, until the samples were submitted for paraffin embedding. With TRIzol reagent and using the manufacturer’s instructions (Invitrogen, Carlsbad, CA, USA), total RNA was isolated from a primary human CRC from its adjacent normal/benign tissue specimen. High-capacity cDNA reverse transcription kits with RNase inhibitor (Applied Biosystems, Thermo Fisher Scientific, Waltham, MA, USA) were used to reverse transcribe RNA. Each reaction was set up with 10 ng cDNA samples in triplicates for RNA endogenous expression of *BCL-2, BCL-X_L_*, and *BCL-W* in normal and CRC samples, using PowerUp SYBR green master mix (Applied Biosystems, Thermo Fisher Scientific, Waltham, MA, USA) on an ABI real-time PCR machine. The RNA expression data were analyzed with Quant-studio real-time PCR software (Applied Biosystems, Thermo Fisher Scientific, Waltham, MA, USA). The primer sequences are summarized in Table 2. Relative fold changes in RNA expression of *BCL-2* family members were calculated using the comparative 2−ΔΔCt method and normalized to the corresponding reference gene (*β-actin*) levels. The Student’s t-test with two-tailed distribution was used to calculate the *p*-values for statistical significance.

### 4.11. TUNEL Staining

To analyze the apoptosis induced by ABT-263, organoids were stained using TACS-2 TdT-DAB In Situ Apoptosis Detection Kits (Cat # 4810-30-K, R&D Biotechne, Minneapolis, MN, USA), following the manufacturer’s instructions.

### 4.12. Immunofluorescence Microscopy

For immunofluorescence staining, 5 µM thick FFPE sections were used. The sections were deparaffinized, rehydrated, and, after antigen retrieval, sections were incubated with BLOXALL (Cat. # SP-6000, Vector Laboratories, Burlingame, CA, USA) for 15 min to remove endogenous peroxidase activity. Tissue sections were then blocked with Normal Horse Serum Blocking Solution (Cat. # S-2012, Vector Laboratories, Burlingame, CA, USA) for 1 h at room temperature and probed with specific antibodies for BCL-2 (Cat # M0887, Agilent, Santa Clara, CA, USA), BCL-X_L_ (Cat # sc-8392, Santa Cruz Biotechnology, Inc. Dallas, TX, USA), BCL-W (Cat #2724, Cell Signaling Technology, Danvers, MA, USA), PUMA (Cat # ab9643, ABCAM, Waltham, MA, USA), cleaved-caspase 3 (Cat # 9661, Cell Signaling Technology, Danvers, MA, USA), or BAX (Cat #89477, Cell Signaling Technology, Danvers, MA, USA) for 1 h at room temperature. After three washes with 0.5% TBST, tissue sections were incubated for 1 h with secondary Alexa-Fluor 546-conjugated antirabbit (BCL-W, PUMA cleaved-caspase 3), secondary Alexa-Fluor 488-conjugated antimouse, (BCL-2, BCL-X_L_, BAX) antibodies. The same sections were costained in the following combinations, (BCL-2-488/PUMA-546), (BCL-W-546/BAX-488), and (BCL-X_L_-488/ cleaved-caspase 3-546), for better comparison of antiapoptotic and proapoptotic proteins. The antiapoptotic proteins (BCL-2, BCL-W, and BCL-X_L_) are presented in Figure 5, and proapoptotic protein markers (PUMA, BAX, and cleaved-caspase 3) are shown in Figure 7. After incubation, sections were washed with 0.5% TBST and mounted with ProLong™ Gold Antifade Mountant containing DAPI (Cat. # P36935, Thermo Fisher Scientific, Waltham, MA, USA). Fluorescence microscopy was performed with a Nikon A1R-HD25 confocal microscope, and images were analyzed using Nis Elements 5.0 Imaging Software.

## Figures and Tables

**Table 1:** Clinicopathologic characteristics of CRCs used in the study.

	CRC Patient #	Sex	Age	Body Mass Index	Sample Origin	Tumor (T), Nodes (N), and Metastases (M)	Degree of Differentiation
1	CRC-18	Female	73	21.92	Colon	T3N0Mx	Moderate
2	CRC-59	Male	40	26.42	Sigmoid Colon	T3N0	Moderate
3	CRC-82	Male	65	34.94	Ascending Colon	T3N1	Moderate

**Table 2:** Primer sequences of transcripts evaluated in the study.

	Genes	Primer Sequence (5′→ 3′)
1	*BCL2*-F	GGA TTG TGG CCT TCT TTG AG
2	*BCL2*-R	GCC GGT TCA GGT ACT CAG TC
3	*BCL-X_L_*-F	AGT TTG AAC TGC GGT ACC GG
4	*BCL-X_L_*-R	GCA TTG TTC CCA TAG AGT TC
5	*BCL W*-F	GCG GAG TTC ACAGCT CTA TAC
6	*BCL W*-R	AAA AGG CCC CTA CAG TTA CCA
7	*β-Actin*-F	CTG CTT GCT GAT CCA CAT CTG
8	*β-Actin*-R	ATC AAG ATC ATT GCT CCT CCT GAG

## Data Availability

All data supporting the reported results are presented in the manuscript.

## References

[1] Drost J., Clevers H. (2018). Organoids in cancer research. Nat. Rev. Cancer.

[2] Ooft S.N., Weeber F., Dijkstra K.K., McLean C.M., Kaing S., van Werkhoven E., Schipper L., Hoes L., Vis D.J., van de Haar J. (2019). Patient-derived organoids can predict response to chemotherapy in metastatic colorectal cancer patients. Sci. Transl. Med..

[3] Sato T., Stange D.E., Ferrante M., Vries R.G., Van Es J.H., Van den Brink S., Van Houdt W.J., Pronk A., Van Gorp J., Siersema P.D. (2011). Long-term expansion of epithelial organoids from human colon, adenoma, adenocarcinoma, and Barrett’s epithelium. Gastroenterology.

[4] Weeber F., van de Wetering M., Hoogstraat M., Dijkstra K.K., Krijgsman O., Kuilman T., Gadellaa-van Hooijdonk C.G., van der Velden D.L., Peeper D.S., Cuppen E.P. (2015). Preserved genetic diversity in organoids cultured from biopsies of human colorectal cancer metastases. Proc. Natl. Acad. Sci. USA.

[5] Meyers B.M., Cosby R., Quereshy F., Jonker D. (2016). Adjuvant systemic chemotherapy for stages II and III colon cancer after complete resection: A clinical practice guideline. Curr. Oncol..

[6] Sobrero A., Lonardi S., Rosati G., Di Bartolomeo M., Ronzoni M., Pella N., Scartozzi M., Banzi M., Zampino M.G., Pasini F. (2018). FOLFOX or CAPOX in Stage II to III Colon Cancer: Efficacy Results of the Italian Three or Six Colon Adjuvant Trial. J. Clin. Oncol..

[7] Aparo S., Goel S. (2012). Evolvement of the treatment paradigm for metastatic colon cancer. From chemotherapy to targeted therapy. Crit. Rev. Oncol./Hematol..

[8] Gonzalo Recondo E.D.-C., de la Vega M., Greco M., Gonzalo Recondo M.E.V. (2014). Advances and new perspectives in the treatment of metastatic colon cancer. World J. Gastrointest. Oncol..

[9] Ramesh P., Medema J.P. (2020). BCL-2 family deregulation in colorectal cancer: Potential for BH3 mimetics in therapy. Apoptosis.

[10] Bedi A., Pasricha P.J., Akhtar A.J., Barber J.P., Bedi G.C., Giardiello F.M., Zehnbauer B.A., Hamilton S.R., Jones R.J. (1995). Inhibition of apoptosis during development of colorectal cancer. Cancer Res..

[11] Reed J. (1994). Bcl-2 and the regulation of programmed cell death. J. Cell Biol..

[12] Manne U., Myers R.B., Moron C., Poczatek R.B., Dillard S., Weiss H., Brown D., Srivastava S., Grizzle W.E. (1997). Prognostic significance of Bcl-2 expression and p53 nuclear accumulation in colorectal adenocarcinoma. Int. J. Cancer.

[13] Corcoran R.B., Cheng K.A., Hata A.N., Faber A.C., Ebi H., Coffee E.M., Greninger P., Brown R.D., Godfrey J.T., Cohoon T.J. (2013). Synthetic lethal interaction of combined BCL-XL and MEK inhibition promotes tumor regressions in KRAS mutant cancer models. Cancer Cell.

[14] Shao H., Jing K., Mahmoud E., Huang H., Fang X., Yu C. (2013). Apigenin Sensitizes Colon Cancer Cells to Antitumor Activity of ABT-263. Mol. Cancer Ther..

[15] Balakrishnan K., Gandhi V. (2013). Bcl-2 antagonists: A proof of concept for CLL therapy. Investig. New Drugs.

[16] Tse C., Shoemaker A.R., Adickes J., Anderson M.G., Chen J., Jin S., Johnson E.F., Marsh K.C., Mitten M.J., Nimmer P. (2008). ABT-263: A potent and orally bioavailable Bcl-2 family inhibitor. Cancer Res..

[17] Chen Q., Song S., Wei S., Liu B., Honjo S., Scott A., Jin J., Ma L., Zhu H., Skinner H.D. (2015). ABT-263 induces apoptosis and synergizes with chemotherapy by targeting stemness pathways in esophageal cancer. Oncotarget.

[18] Rysanek D., Vasicova P., Kolla J.N., Sedlak D., Andera L., Bartek J., Hodny Z. (2022). Synergism of BCL-2 family inhibitors facilitates selective elimination of senescent cells. Aging.

[19] Place T.L., Domann F.E., Case A.J. (2017). Limitations of oxygen delivery to cells in culture: An underappreciated problem in basic and translational research. Free Radic. Biol. Med..

[20] Kety S.S. (1951). The theory and applications of the exchange of inert gas at the lungs and tissues. Pharmacol. Rev..

[21] Krogh A. (1919). The rate of diffusion of gases through animal tissues, with some remarks on the coefficient of invasion. J. Physiol..

[22] Peniche Silva C.J., Liebsch G., Meier R.J., Gutbrod M.S., Balmayor E.R., van Griensven M. (2020). A New Non-invasive Technique for Measuring 3D-Oxygen Gradients in Wells During Mammalian Cell Culture. Front. Bioeng. Biotechnol..

[23] Al-Ani A., Toms D., Kondro D., Thundathil J., Yu Y., Ungrin M. (2018). Oxygenation in cell culture: Critical parameters for reproducibility are routinely not reported. PLoS ONE.

[24] Wenger R.H. (2000). Mammalian oxygen sensing, signalling and gene regulation. J. Exp. Biol..

